# Parent Emigration, Physical Health and Related Risk and Preventive Factors of Children Left Behind: A Systematic Review of Literature

**DOI:** 10.3390/ijerph18031167

**Published:** 2021-01-28

**Authors:** Justina Račaitė, Jutta Lindert, Khatia Antia, Volker Winkler, Rita Sketerskienė, Marija Jakubauskienė, Linda Wulkau, Genė Šurkienė

**Affiliations:** 1 Department of Public Health, Institute of Health Sciences, Faculty of Medicine, Vilnius University, M.K. Čiurlionio Str. 21, LT-03101 Vilnius, Lithuania; rita.sketerskiene@mf.vu.lt (R.S.); marija.jakubauskiene@mf.vu.lt (M.J.); gene.surkiene@mf.vu.lt (G.Š.); 2 Department of Social Work and Health, University of Applied Sciences Emden/Leer, Constantiaplatz 4, 26723 Emden, Germany; jutta.lindert@hs-emden-leer.de (J.L.); linda.wulkau@hs-emden-leer.de (L.W.); 3 WRSC, Brandeis University, Epstein Building, MS 079, 515 South Street, Waltham, MA 02453, USA; 4 Heidelberg Institute of Global Health, Heidelberg University Hospital, Im Neuenheimer Feld 130/3, 69120 Heidelberg, Germany; khatia.antia@uni-heidelberg.de (K.A.); volker.winkler@uni-heidelberg.de (V.W.); 5 Institute for Epidemiology, Social Medicine and Health Systems Research, Hannover Medical School, Carl-Neuberg-Straße 1, 30625 Hannover, Germany

**Keywords:** children left behind, parental migration, physical health, children health

## Abstract

The aim of our study was to systematically review the literature on physical health and related consequences of internal and international parental migration on left-behind children (LBC). This review followed PRISMA guidelines. We searched the PubMed, Web of Science, Academic Search Complete, PsycINFO, and Cochrane databases and included studies reporting physical health-related outcomes of children affected by parental migration. The quality of the studies was assessed using the Quality Assessment Tool for Observational Cohort and Cross-Sectional Studies. We selected 34 publications from a total of 6061 search results. The study found that LBC suffer from poor physical health as compared with non-LBC. Physical health-related risk factors such as underweight, lower weight, stunted growth, unhealthy food preferences, lower physical activity, smoking, alcohol consumption, injuries, and incomplete vaccination tend to be more prevalent among LBC in China. Studies focussing on international migration argue that having migrant parents might be preventive for undernutrition. Overall, our study showed that children affected by internal or international migration tend to have similar physical health outcomes. Moreover, we identified a lack of evidence on international parental migration that may have influenced the overall impacts. Further studies addressing international migration would contribute to better understand the impacts of migration for LBC.

## 1. Introduction

The United Nation’s Convention on the Rights of the Child “ [recognized] that the child, for the full and harmonious development of his or her personality, should grow up in a family environment, in an atmosphere of happiness, love and understanding” [1]. The attachment theory, formulated by John Bowlby, states that for the successful social and emotional development, every child needs a close relationship with at least one primary caregiver [2]. However, there are multiple reasons for parental absence, such as divorce or death. Moreover, children are sometimes taken from unsafe family environments temporarily or permanently.

The migration of parents is another form of child separation from one or both parents. The International Organization for Migration defines migration as “the movement of a person or a group of persons, either across an international border, or within a State [independent of] its length, composition and causes [including] migration of refugees, displaced persons, economic migrants, and persons moving for other purposes” [3]. The World Migration Report has reported that, in 2019, the number of international migrants globally was 272 million, which is around 3.5% of the world’s population [4]. Migration is important for the economic growth and improvement of countries. The overwhelming majority of people choose to migrate internationally for reasons related to work, family, and study. However, events such as a conflict, persecution, and disaster force people to migrate without having any choice [4]. Safe, orderly, and regular international migration is included in the 2030 Agenda for Sustainable Development Goals [5].

Employment migration may cause a number of short- and long-term consequences. One of them is the separation of families where children are left behind in their region or countries of origin. In the available literature, children left behind are defined as individuals below the age of 18, whose parent(s) migrate to other places for work for at least six months [6,7]. Thousands of children are considered to be left behind in many low- and middle-income countries. For example, it has been estimated that in the Philippines, 27%, Ecuador, 36%, and rural South Africa, 40% of children have at least one migrant parent [8].

Health issues of left-behind children are increasingly being discussed in the scientific literature. Some authors have conducted systematic literature reviews focussing mostly on rural–urban migration in China [8,9,10,11]. Previous studies in this field have made an important contribution regarding the understanding of how parental migration affects the social environment and psychological well-being of LBC, education, and health [8,12,13,14]. Following scientific interest, the United Nations Children’s Fund (UNICEF) recently drew attention to the vulnerability of these children [15]. This issue has been well explored in China, where migration happens internally, from rural to urban areas. Despite the increased attention, there is a lack of data on the impact of international parental migration on LBC’s physical health outcomes. Our study seeks to analyse and synthesize the most recent evidence on health consequences of internal and international parental migration on LBC’s physical health and related risk and preventive factors.

## 2. Materials and Methods

For this study, we followed the Preferred Reporting Items for Systematic Reviews and Meta-Analyses (PRISMA) guidelines [16].

We searched PubMed, PsycINFO, Web of Science, Academic search complete, and Cochrane databases for relevant studies published up to 15 May 2020. Our search was guided by the concept of population, exposure, comparator, and outcomes (PECO). We applied the following search terms: left alone OR left behind and stay at home OR left over AND child* AND parent* AND emigrant* OR migrant household AND physical* health OR overweight OR obesity OR stunting OR vaccination OR breastfeed* OR “physical* activity”. In addition, we searched the reference lists of included studies and relevant systematic reviews.

We included studies based on the following criteria: (1) study population children (below age 18), (2) original study, (3) one or both parents live in internal or international migration, (4) quantitative measure of physical health outcomes on children, (5) available in English. Two authors (J.R. and R.S.) independently performed title and abstract screening. Disagreements were solved by discussions with a third opinion (G.Š.). Studies with the following criteria were excluded: published before 1 January 2008; qualitative or experimental studies; mental health, well-being or educational outcomes; and children living in migration together with parents.

Two authors (J.R. and R.S.) extracted the following information from included papers: first author; year of publication; geographical area; study design; sample size and method, age and gender distribution, definitions and measures of exposures and outcomes, results, covariates and limitations. Disagreement was solved by including a third opinion (G.Š.).

In this systematic review, we analysed studies from the following two perspectives: (1) type of migration (internal/international) outcomes and (2) physical health outcomes and related risk and preventive factors (weight and height, nutrition, health behaviour, injuries, immunization).

The quality of the studies was assessed using the Quality Assessment Tool for Observational Cohort and Cross-Sectional Studies (National Heart, Lung, and Blood Institute, 2014). Assessment consisted of 14 questions. Studies were defined as “good”, “fair”, or “poor” according to the number of “yes” answers in the evaluation from 50%, 49–21%, and below 20%, respectively. Two reviewers (J.R. and L.W.) independently rated the studies and disagreements were resolved by consensus including a third opinion (G.Š.).

## 3. Results

### 3.1. Study Selection and Characteristics

**Figure 1:**
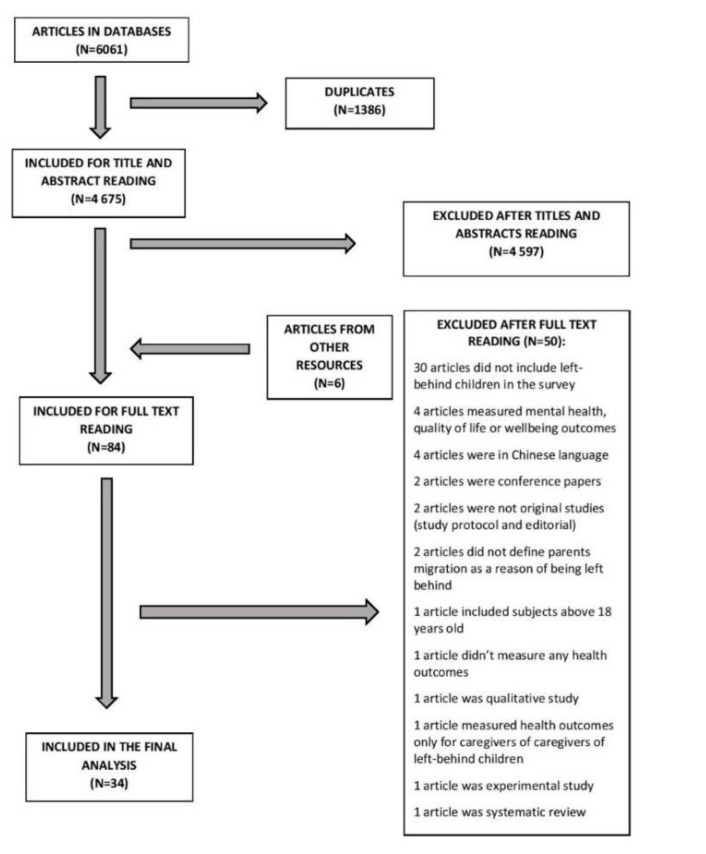
Study selection scheme.

We identified 6061 studies by searching the databases, from which 1386 were duplicates. We excluded 4597 records after title and abstract screening. Six articles were added after searching the reference lists of included studies. Full-text reading of 84 articles led to the exclusion of 50 manuscripts. We included 34 studies published between 1 May 2008 and 12 March 2020 in the final analysis. Reasons of exclusion are given in Figure 1.

Included studies were cross-sectional (26) or longitudinal (8). The majority of studies (25) were conducted in China. Studies conducted in other countries included the following: two studies from Mexico [17,18]; two studies from Sri Lanka [19,20]; one study from the Philippines [21]; one study from Bangladesh [22]; one study from the Philippines and Vietnam [23]; one study from Ethiopia, India, Peru, and Vietnam [24]; one study from Moldova and Georgia [25]. We evaluated 30 studies as “good” and four studies as “fair”. The detailed characteristics of included studies are provided in Table 1. Table 2 describes the main outcome measures. 

### 3.2. Internal Migration and Physical Health Outcomes

In China, five studies found that parental migration had negative consequences on child health [32,33,35,42,43]. As compared with children of non-migrant parents, LBC were more susceptible to illness and had a higher prevalence of acute and chronic diseases [33,35]. Being a child left behind was also strongly and positively associated with the pre-hypertension or hypertension (OR = 7.77, *p* < 0.01) [43].

Studies included in our analysis reported contradictory results with respect to physical health outcomes of LBC with one or both migrant parents. In China, findings suggest that a mother’s absence alone would not affect a child’s health, but both a mother’s and father’s absence together would have a significant negative effect on LBC in rural China [33]. Children raised by a single parent tend to be more susceptible to illness than children raised by both parents [35]. Among LBC, those living with their mother were more likely to be in better health, than those living with their father only [33].

Regarding the age and a gender of LBC, some studies suggested that left-behind adolescents (13–18 years) could have worse outcomes than younger children [7]. Girls may be more vulnerable than boys to the absence of parental care [33].

One study did not find any significant relationship between parent emigration and child health status [28] and one study found no evidence that children living in migration together with their parents had better health than children left behind [7].

### 3.3. Internal Migration and Risk and Preventive Factors

#### 3.3.1. Nutrition, Weight, and Height

Studies included in this review found that, in China, parental migration negatively affected child nutrition [45]. Firstly, as compared with the control group (children of non-migrant parents), children left behind were less likely to receive age-appropriate breastfeeding and the duration of breastfeeding was significantly shorter [26,34]. Total food intake, as well as intake of meat, fish and eggs were lower among LBC [34]. Moreover, children with both parents absent were most likely to skip breakfast, as well as eat high-fat food and sweetened snacks [6,46]. Higher fat and lower protein diet were more common among left-behind boys [46]. Accordingly, more LBC disliked vegetables (M = 3.66, SD = 0.55) and fruits (M = 3.81, SD = 0.47, *p* < 0.01) than non-LBC (M = 3.89, SD = 0.27 and M = 3.97, SD 0.83, *p* < 0.01) [40]. Due to iron-poor food intake, LBC were at a higher risk of developing anaemia, especially at a younger age [29,34]. Additionally, Zhang et al. found gender differences, for example, on the one hand, LB boys in early childhood showed slower height and weight gain as compared with boys living in non-migrant households [47]. On the other hand, Gao et al. suggested that children left behind might have an increased risk of being overweight [6]. 

Two studies found no negative impact of parental migration for weight and height outcomes [34,41]. One study found high rates of nutrition problems regardless of parental migration status [48].

#### 3.3.2. Unhealthy Behaviours

In general, children with both parents absent were more likely to engage in risky behaviours such as unhealthy diet, physical inactivity, sedentary lifestyle, smoking, and drinking [48].

With respect to addictive behaviours, studies found that alcohol consumption and smoking were higher among children of both migrant parents as opposed to only one (or none) migrant parent [6,31,44]. Other authors claimed that maternal migration increased the risk of adolescent smoking, while paternal migration could even protect children from smoking [27]. In terms of gender, some authors found that LB girls were at a higher risk for smoking and binge drinking [6], whereas other studies reported that more LB boys tended to be smokers and current alcohol users than LB girls [31,44].

#### 3.3.3. Injuries

Some authors have suggested that LBC have a higher risk of getting injured [30,37]. The annual injury rate was more than double among LBC as compared with children living with both parents [37]. When controlling for other variables, LBC were more likely to experience unintentional injuries than residential children [30]. The most frequently reported injuries were falls, contact with sharp instrument, striking by objects or person, bitten, or struck by animals, and injuries caused by nature or environment factors [30].

#### 3.3.4. Immunization

Studies included in this review found a lower coverage of vaccination among LBC as compared with non-LBC [38]. Children of non-migrating parents (95.7%) were more likely to receive complete vaccination as opposed to LBC (79.9%, *p* < 0.001) [36]. Moreover, LBC had significantly lower coverage of timely vaccination [39].

### 3.4. International Migration and Physical Health Outcomes

Physical health outcomes were analysed in three studies conducted in different regions [17,21,25]. A study from the Philippines found that left-behind adolescents (M = 5.09, SD = 0.78) reported poorer physical health than non-LBC (M = 5.43, SD = 0.63), *p* < 0.01). The same study found that left-behind adolescents (13–18 years) might be more negatively affected than younger children [21].

A Mexican study compared differences between mother and father emigration and found poorer health outcomes among LBC of international migrant fathers then children of non-migrant fathers [17].

One study conducted in Moldova and Georgia did not find any significant association between parent emigration and child health status [25].

### 3.5. International Migration and Risk and Preventive Factors

#### 3.5.1. Nutrition, Weight, and Height

Regarding weight and height outcomes, findings from Sri Lanka found a lower prevalence of stunting (11.5% vs. 14.8%), wasting (18.1% vs. 21.5%), and underweight (24.3% vs. 26.2%) among LBC as compared with non-LBC, respectively [19]. A study from Ethiopia, India, and Peru found higher weight and height and lower proportion of malnourished children in migrant households as opposed to children from non-migrant households [24]. However, another study conducted in the Philippines and Vietnam did not support this [23].

Two studies from Bangladesh and Sri Lanka found no negative impact of parental migration for weight and height outcomes [20,22].

#### 3.5.2. Physical Activity

There was only one study from Mexico which found lower physical activity among LBC than non-LBC. Children with parental migration experience had 0.56 less physical active time (hours) per day as compared with children from non-migrant households [18].

## 4. Discussion

In this study, we systematically reviewed the evidence on the effects of internal and international parental migration on their children’s physical health outcomes and related risk and preventive factors. By doing so, we provided comparative analysis of the outcomes of the internal rural–urban and international migration. Previous studies in this field analysed the outcomes independently from type of migration [8] or focused on international migration only [14]. Our study was motivated by the substantial research gap in research on international labour migration effects on LBC in many low- and middle-income countries. A predominance of studies focused on internal migration from China have clearly shown the emerging need to shift this paradigm and investigate the issue in a global context [49].

As explained above, all studies analysing internal migration outcomes were conducted in China, while all studies from other regions (Americas, South and Southeast Asia, Africa, and the East European Region) examined international parental migration outcomes on LBC. Our study found that, despite the type of migration/region, LBC suffer from poor general health. Children with migrating parents are at a higher risk of developing poor nutrition, overweight or obesity, addictive behaviours, physical inactivity, lower vaccination coverage, and more frequent injuries than non-LBC. Some authors have explained such findings using the cognitive stress theory developed by Lazarus and Folkman, in 1984 [21]. Our findings are consistent with previous studies [8] showing that LBC’s physical health outcomes are not improving over time.

Despite the negative outcomes reported, several authors also discuss the potential benefits of parental migration on their children’s physical health. Some studies suggested that remittances could prevent undernutrition and improve access to medical care for LBC [17,19,25]. In contrast, studies that focused on internal migration from China found that remittances were related to a higher risk of overweight [35].

Studies included in this review found that socioeconomic conditions and characteristics of caregivers play an important role for potential outcomes. The following factors were most reported: parents’ and caregivers’ education, sex of migrant parent, household size, income per-capita, parental marital status, and siblings. The health literacy of a primary caregiver was found to be essential for nutrition, health, and development of a child [35]. Our study findings show that having a migrant mother might be more harmful, than having a migrant father [6,19,33]. Some authors emphasize the negative influence of a culture, for example, traditions of physical punishments in some countries, such as Moldova and Georgia [50]. However, most of the outcomes stay negative and significant after controlling for potential socioeconomic cofounders (Table 2).

In general, studies from China and Mexico found that LBC might be more vulnerable to risky behaviour and an unhealthy lifestyle than non-LBC. LBC affected by internal migration tend to have more risky behaviour such as alcohol consumption, smoking, as well as a high fat and low protein diet [44,47]. A study from Mexico also found lower physical activity among LBC [18]. This shows the need for improving health literacy and health education in schools and among caregivers of LBC.

Several limitations of our study should be mentioned. We included studies published only in English. Most of the included articles focused on Chinese populations, with a few exceptions from the Caribbean region, South America, South Asia, and Europe. Among all the included studies, only nine studies focused on international migration, while all other studies came from China, and therefore addressed only internal migration. Most of the studies were cross-sectional, which did not allow drawing conclusions on causation. Finally, authors noted that according to the International Organization of Migration, labour migration trends have increased significantly in recent years [4]. With this in mind, we decided to included studies published after 1 January 2008 aiming at providing the most recent evidence in this field. However, this could be considered to be a limitation, since we may have missed some relevant studies. Despite these limitations, to the best of our knowledge, this is the first study considering both internal and international migration aspects while examining the effects of parental migration on LBC’s physical health.

The importance of migration is growing together with globalization, while millions of children are left behind in their countries of origin. Our findings emphasize the need for preventive actions to address the health of LBC. Various international organizations such as UNICEF have brought attention to this vulnerable groups. Even though scholars have addressed this issue in China, there is an urgent need for more evidence from other labour migration-affected regions of the world. Public health interventions for LBC is needed.

## 5. Conclusions

This study found that both internal and international parental migration is associated with child outcomes such as physical health, nutrition, weight and height, injuries, and immunization. In most cases, the consequences for child health are negative, however, in low- and middle-income countries parental migration might also prevent left-behind children from undernutrition. When comparing studies from China and other countries, we found similar outcomes (regardless of internal or international migration). This study highlights the knowledge gap on the topic, especially in Western Asia and the East European Region, and calls for action from governments and international institutions, and the research community to better investigate and address the health needs of children affected by parental migration.

## Figures and Tables

**Table 1:** Characteristics of the studies.

No	First Author, Year of Publication	Country	Study Design	Sample Size (N)	Age (Range, Mean, SD)	Gender Distribution (Male; %)	Outcomes	Quality Rating ^1^
1.	Ban, 2017 [26]	China	Cross-sectional	6136	0–35 months	Neither parent migrated 54.3%, father migrated only 52.7%, mother with/without father migrated 54.2%	1. Stunting 2. Breastfeeding 3. Milk feeding	Good
2.	Cebotari, 2018 [25]	Moldova, Georgia	Cross-sectional	Moldova (1601), Georgia (1193)	10–18 years Moldova 14.3 (SD 2.59), Georgia 13.44 (SD 2.40)	Moldova 51.93%, Georgia 53.96%	Child health status	Good
3.	Edelblute, 2019 [17]	Mexico	Cross-sectional	542	5.3 (SD 2.96)	Father present 51.5%, father absence (migration) 48.1%, father absence (other reasons) 42.9%	Maternal ratings of child poor health	Good
4.	Gao, 2013 [27]	China	Cross-sectional	2558	13.8 (SD 1.14)	55%	1. Past 30 days smoking 2. Self-efficacy of smoking	Good
5.	Gao, 2010 [6]	China	Cross-sectional	2986	10–18 years 14.2 (SD 1.4)	51.4%	1. Health-related behaviours 2. Nutritional status	Good
6.	Graham, 2013 [23]	The Philippines, Vietnam	Cross-sectional	The Philippines (480), Vietnam (482)	9–11 years	NA ^2^	Stunting	Good
7	Guo, 2017 [28]	China	Longitudinal	6083	12.27 (SD 3.71)	55%	Self-rated health	Good
8.	Hipgrave, 2014 [29]	China	Cross-sectional	2244	6–23 months	56.3%	Haemoglobin concentration	Good
9.	Hu, 2018 [30]	China	Cross-sectional	4479	6–16 years	46.5%	Unintentional injuries	Good
10.	Huang, 2018 [7]	China	Cross-sectional	916	11.6	57%	Children’s health conditions	Good
11.	Islam, 2019 [22]	Bangladesh	Cross-sectional	23,402	0–5 years 2	51.3%	1. Stunting 2. Wasting 3. Underweight 4. Nutritional disorders	Good
12.	Jayatissa, 2016 [19]	Sri Lanka	Cross-sectional	7500	6–59 months 32.9 (SD 14.7)	50.2%	1. Stunting, wasting and underweight 2. Health status 3. Food intake	Fair
13.	Jiang, 2015 [31]	China	Cross-sectional	1367	12.2 (SD 1.3)	56%	Current alcohol drinking	Good
14.	Lei, 2018 [32]	China	Cross-sectional	5413	1–15 years	Rural without migrant parents 55%;left behind 56%.	1. Child health status 2. Child health status assessed by guardians	Good
15.	Li, 2015 [33]	China	Longitudinal	13,171	9755 (SD 4.9)	53%	1.Sickness 2. Injuries 3. Chronic conditions 4. Acute disease	Good
16.	Luo, 2008 [34]	China	Cross-sectional	1548	0.33–6.92 years 3.51 (SD 1.59)	56.2%	1. Anthropometry 2. Dietary intake 3. Haemoglobin concentration	Fair
17.	Mo, 2016 [35]	China	Cross-sectional	735	1–6 years 4.58 years (SD 55.0 months)	44.1%	Physical health	Good
18.	Nguyen, 2016 [24]	Ethiopia, India, Peru, Vietnam	Cross-sectional	7725	5–8 years	NA^2^	Health status	Good
19.	Ni, 2017 [36]	China	Cross-sectional	1368	12–72 months	85%	1. The full vaccination rate 2. The age-appropriate vaccinationrate	Good
20.	Palos-Lucio, 2015 [18]	Mexico	Cross-sectional	239	9–12 years	Migrant household 51.69%; Non-migrant household 55.46%.	1. Physical activity	Good
21.	Shen, 2009 [37]	China	Cross-sectional	3019	5–17 years	60.3%	Unintentional injuries	Fair
22.	Smeekens, 2012 [21]	The Philippines	Cross-sectional	205	13–18 14.58 (SD 1.04)	31.7%	Physical health	Good
23.	Tang, 2019 [38]	China	Cross-sectional	1662	12–15 years	50.2%	1. Physical health status 2. Health behaviours 3. Frequency of not going to school due to sickness 4. Vaccination	Good
24.	Tang, 2016 [39]	China	Cross-sectional	1216	18–24 months	50.2%	Vaccination status	Good
25.	Tao, 2016 [40]	China	Cross-sectional	827	7–15 years	51.2%	1. Nutritive status 2. Food preference	Good
26.	Tian, 2017 [41]	China	Longitudinal	446	11–18 years	60%	1. Growth 2. Nutrition	Good
27.	Tong, 2015 [42]	China	Longitudinal	8662	10.18 (SD 4.77)	83.8%	Childhood illness	Good
28.	Wen, 2016 [43]	China	Longitudinal	2170	7–17 years	At baseline 53.9%	Blood pressure	Good
29.	Wickramage, 2015 [20]	Sri Lanka	Cross-sectional	770	1–17 years	Left-behind group 50.6%; control group:53.2%.	1. Nutritional status 2. Anthropometry 3. Immunization history	Good
30.	Yang, 2016 [44]	China	Cross-sectional	1343	10–14 years	56%	Smoking	Good
31.	Yue, 2020 [45]	China	Longitudinal	1802	6–30 months	53.1%	1. Nutritional status 2. Anaemia status 3. General health 4. Feeding practises	Good
32.	Zhang, 2015 [46]	China	Longitudinal	2555	0–17 years	57.3%	Child growth	Good
33.	Zhang (a), 2015 [47]	China	Longitudinal	975	1–17 years	56.9%	1. Dietary 2. Macronutrient intakes	Good
34.	Zhou, 2015 [48]	China	Cross-sectional	141,000	3–17 years	NA ^2^	1. Health 2. Nutrition	Fair

^1^ Quality of the studies assessed using National Institute of Health Quality Assessment Tool for Observational Cohort and Cross-Sectional Studies. Possible quality ratings (good, fair, poor). ^2^ NA, no data available.

**Table 2:** Main outcomes (only statistically significant outcomes provided).

Group	Outcome (Left-Behind Children)	Sample Size (N)	Covariates ^2^	Statistics (OR, Mean, SD, *p*, 95% CI or Other Statistics If Provided) ^1^	Reference
**Internal Migration**					
**Physical Health**	More susceptible to illness	735	Parenting styles, age of child, health literacy	OR 1.628, *p* < 0.05	Mo, 2015 [35]
	Childhood illness	8662	Age, gender, household size, income per capita, grandparents living together, village context, village size	OR 1.29, SE = 0.164, *p* < 0.05	Tong, 2015 [42]
	Pre-hypertension or hypertension	2170	Age, gender, mothers education, fathers education, annual household per capita income	OR 7.77, 95% CI 2.05–29.4, *p* < 0.01	Wen, 2016 [43]
**Nutrition, Weight and Height**	Lower Height for age z-score	5413	Age, gender	OR −0.165, *p* < 0.01	Lei, 2018 [32]
	Lower Weight for age z-score	5413	Age, gender	OR −0.142, *p* < 0.05	Lei, 2018 [32]
	Malnutrition rate	827	NR/NA	LBC 14.83%, NoN-LBC 7.04%, *p* < 0.01	Tao, 2016 [40]
	Less likely to be ever breastfed	6136	Age, gender, ethnicity, elder siblings, guardian education attainment, the number of household electrical appliances, year of survey	OR 0.30, 95% CI 0.17–0.52	Ban, 2017 [26]
	Less likely to be breastfed	1548	NR/NA	LBC (78.7%), Non-LBC (82.8%), *p* < 0.05	Luo, 2008 [34]
	Shorter breastfeeding	6136	Age, gender, ethnicity, elder siblings, guardian education attainment, the number of household electrical appliances, year of survey	β-3.77, 95% CI −5.01–−2.53	Ban, 2017 [26]
	Duration of breastfeeding (months)	1548	NR/NA	LBC M = 9.48, SD = 3.58; Non-LBC M = 10.70, SD = +3.26; *p* < 0.001	Luo, 2008 [34]
**Unhealthy behaviors**	Alcohol use	1367	Gender, age, grade, if only child in the family, perceived school performance	OR 2.01, 95% CI 1.28–3.16, *p* < 0.05	Jiang, 2015 [31]
	Higher smoking rate	1343	Gender, grade, if only child in the family, perceived school performance	OR 5.59, 95% CI 2.38–13.15, *p* < 0.001	Yang, 2016 [44]
**Injuries**	More likely to experience injury	4479	Gender, age, fair physical health, school academic achievement, only child in the family, household income level, parental marital status; maternal education attainment, family conflicts; model school; peer rejection, rural region	OR 1.208, SE 0.104, *p* < 0.05	Hu, 2018 [30]
	Higher injury rate	3019	NR/NA	LBC 252.9/1000, 95% CI 233.0–273.0, Non-LBC 119.7/1000, 95% CI 104.9–134.7, *p* < 0.0001	Shen, 2009 [37]
**Immunization**	Lower rates of timely vaccination	1216	NR/NA	LBC55.7%, 95% CI 51.3–60.0, Non-LBC 60.8, 95% CI 57.3–64.0, *p* = 0.011	Tang, 2016 [39]
	Less likely to receive complete vaccination	1368	NR/NA	LBC 92.7%, Non-LBC 79.9%, X^2^ = 35.2, *p* < 0.001	Ni, 2017 [36]
	Lower coverage of complete vaccination	1662	NR/NA	LBC 38.7%, Non-LBC 44.2%, *p* < 0.036	Tang, 2019 [38]
**International Migration**					
**Physical Health**	Maternal reported child poor health	542	Data from model with no covariates added	OR 0.33, 95% CI 0.16–0.7, *p* < 0.01	Edelblute, 2018 [17]
	Poorer physical health	205	NR/NA	LBC M = 5.09, SD = 0.78; NoN LBC M = 5.43, SD = 0.63; F (1.199) = 9.08; *p* < 0.01	Smeekens, 2012 [21]
**Unhealthy behaviors**	Lower physical activity	239	Age, gender, body mass index, maternal schooling, paternal schooling, household characteristics	OR −0.56, *p* < 0.05	Palos-Lucio, 2015 [18]

^1^ LBC, left-behind children; Non-LBC, not left-behind children; ^2^ NR/NA, not reported or not applicable.
